# Competitive Game Play Attenuates Self-Other Integration during Joint Task Performance

**DOI:** 10.3389/fpsyg.2016.00274

**Published:** 2016-03-03

**Authors:** Margit I. Ruissen, Ellen R. A. de Bruijn

**Affiliations:** ^1^Department of Clinical Psychology, Leiden UniversityLeiden, Netherlands; ^2^Leiden Institute for Brain and Cognition, Leiden UniversityLeiden, Netherlands

**Keywords:** social Simon effect, self-other integration, social interaction, cooperation, competition

## Abstract

Joint task performance is facilitated by sharing and integrating each other’s action representations. Research has shown that the amount of this so-called self-other integration depends on situational aspects related to the social context, including differences in the social relationship between co-acting individuals. There are indications that a cooperative relationship facilitates self-other integration while a competitive relationship results in more individualistic task performance. However, findings from previous studies in which the cooperative or competitive element was manipulated during task performance are inconsistent. Therefore, the present study aimed to manipulate the social relationship between two individuals prior to performing a social Simon task. This task is frequently used to measure self-other integration and distinction processes. A mixed-within-and-between-subjects design was used in which three groups of participants performed both a standard Simon task and a social Simon task after having played a Tetris game either individually, in cooperation with a co-actor, or in competition against another participant. Performance on the standard Simon task was not affected by the Tetris manipulation. However, a sustained effect of the induced cooperative versus competitive relationship was found on the social Simon Task. Less self-other integration was found in participants who had first played a competitive Tetris game compared to participants who had played a cooperative or solo version of the game. The current study thus demonstrates that an established cooperative or competitive relationship is sufficient to modulate the degree of self-other integration on subsequent joint task performance. Importantly, by using Tetris, attention to others’ actions was beneficial both during cooperative and competitive game play and can thus not explain the competition-induced reduction of self-other integration.

## Introduction

Interacting with other people is an important part of everyday life. In many daily activities, we perform a task together with another person such as a friend, spouse, colleague, or even a complete stranger. To successfully accomplish this, many processes are involved. We need to, for example, distinguish between our own actions and others’, but also coordinate and integrate these actions accordingly. When performing a task jointly with another person, a cognitive representation of the actions and/or tasks of the other person is thought to facilitate successful joint performance (Sebanz et al., 2006; de Bruijn et al., 2011). The notion that people share each other’s action representations during joint action is in line with concepts postulated in the theory of event coding (Hommel et al., 2001) and the ideomotor theory (Prinz, 1997). According to these theories, all actions are cognitively represented in terms of its action consequences. Observing someone perform an action activates the same cognitive representations as when we perform the action our self. Subsequently, when performing a task together with another person, a representation of the other’s actions is automatically integrated into our own task representation, a process also known as self-other integration (see, e.g., Colzato et al., 2012).

Evidence that self-other integration is in general an automatic process comes from studies using a social version of the Simon paradigm in which the Simon task is shared between two individuals (Sebanz et al., 2003). In a standard Simon task (Simon and Rudell, 1967) participants have to respond to, e.g., the color of a stimulus presented left or right of a fixation cross by pressing one of two spatially located buttons. Due to automatic coding of the spatial location of stimuli and response buttons, task performance is facilitated when stimulus and response location are compatible but not when stimulus and response location are incompatible (Lu and Proctor, 1995). The difference in mean reaction time between compatible and incompatible trials is commonly referred to as the Simon effect. Interestingly, a similar compatibility effect is observed when the Simon task is distributed between two individuals. In this so-called social Simon task, two participants seated next to one another each respond to one of the two spatially presented stimuli, thus resulting in participants performing two complementary Go/NoGo tasks. As in the standard Simon task, people in this social version of the task also respond faster when stimulus and response location are compatible than when stimulus and response location are incompatible. In this case, the difference in reaction time on compatible and incompatible trials is referred to as the social Simon effect (SSE; Sebanz et al., 2003). The SSE is thought to follow from the integration of a cognitive representation of the co-actors task into one’s own task representation (Sebanz et al., 2006; Dolk et al., 2014). According to the referential coding account by Dolk et al. (2014), the SSE emerges as a result of the perceived similarity between the action consequences of one’s own task (e.g., a left button being pressed) and the action consequences of the co-actor’s task (e.g., a right button being pressed). The spatial dimension distinguishes between the two action representations and is therefore needed as a reference to discriminate between the two action alternatives. As with the standard Simon effect, the SSE results from using this spatial dimension as a reference and is thus interpreted as reflecting the extent to which people integrate the action representations of another co-actor into their own cognitive representation during joint task performance. The SSE derived from the social Simon task is therefore often used as a measure for self-other integration and distinction processes (see, e.g., van der Weiden et al., 2016).

Recent research has shown that the amount of this self-other integration may be modulated by state variables including the relationship between actor and co-actor. For instance, individuals performing the social Simon task with a positive friendly acting co-actor showed a larger SSE than individuals performing the task with a negative unfriendly co-actor (Hommel et al., 2009). This implies that a positive relationship with the co-actor enhances self-other integration while a negative relationship with the co-actor results in less self-other integration. Also taking the perspective of the co-actor has been shown to enhance self-other integration (Müller et al., 2011b). Since a positive relationship or the ability to take the perspective of a co-actor are prerequisites for efficient cooperative behavior, we expect enhanced self-other integration when cooperating with another agent. Whether, on the other hand, self-other integration is beneficial in a competitive context may depend on contextual factors such as whether the task of a co-actor conflicts with one’s own task or not. Previous studies have investigated how the cooperative or competitive nature of the relationship between two individuals affects self-other integration by giving a monetary reward to the best performing couples (cooperative condition) or the best performing participant of a couple (competitive condition) depending on their performance during the social Simon task (Ruys and Aarts, 2010; Iani et al., 2011). While these previous studies have shown that cooperation indeed seems to enhance self-other integration, findings from the competitive conditions are inconsistent. Iani et al. (2011) found a SSE when participants had to cooperate but not when participants had to compete against one another. Ruys and Aarts (2010), on the other hand, found shared representations both in a cooperative and competitive context. Importantly, these studies differed with respect to both the tasks (the use of visual versus auditory stimuli) and the setting (participants performed the task next to each other in the same room or in different adjacent rooms). When manipulating the social relationship between two individuals during performance of the social Simon task, such variances in task, setting, instructions, and individual differences in the interpretation of the instructions may have resulted in different outcomes. These contextual differences may for instance affect self-other integration by divergent effects on attentional processes. In certain situations it might be beneficial to always attend to the co-actors performance, for instance when one can learn from the decisions and mistakes of others (de Bruijn et al., 2009, 2012). However, in the social Simon task, where integrating the co-actors task results in interference with one’s own task, performance is actually optimized by not attending to the co-actor’s actions. Performance on the task may then thus importantly depend on contextual differences such as the co-actor being in the same room or in another adjacent room. Moreover, individual differences in motivation and attentional focus may affect self-other integration. While people are likely to maintain attention to the co-actor during cooperation, e.g., as a result of bonding, people may actually disengage from their opponent or the task because they are not motivated to compete or because they feel that they are losing the competition. de Bruijn et al. (2008) showed for instance that sharing of action representations only impaired performance of unsuccessful competitors while successful competitors were able to refrain from integrating the action representation of the co-actor. Depending on these variations, performing a task in a competitive setting may thus in certain contexts or individuals result in more attention for one’s own task, while in other contexts people may also attend to the competitor’s task. Because such contextual factors may, at least partly, explain previous findings, it remains unclear whether cooperative or competitive relationships differently affect the extent to which people share each other’s task during performance of the social Simon task.

To prevent possible effects of contextual and motivational factors, the aim of the current study was to put people into a cooperative or competitive relationship *before* jointly performing a *neutral* social Simon task. By using the well-known computer game of Tetris as a way of manipulation before task performance, we made sure that the amount of attention toward own and other’s actions during the cooperative and competitive context manipulation was matched. Different studies have shown that state induction prior to performance of the social Simon task (Kuhbandner et al., 2010; Colzato et al., 2013) may affect self-other integration. In these studies, a negative or self-oriented induction reduced the SSE whereas a positive or socially oriented induction enhanced the SSE. We expect similar sustained modulating effects of established cooperative and competitive relationships on performance of a neutral social Simon task. Moreover, since studies using different methods have suggested that competing with another person involves less integration of the self and other (Decety et al., 2004) whereas cooperating with another person is associated with increased self-other merging (De Cremer and Stouten, 2003), reduced SSEs are expected following competitive game play compared to cooperation.

## Materials and Methods

### Participants

One hundred and sixteen undergraduate students from Leiden University participated in the study. Participants were randomly assigned to one of three conditions. Data from two participants were excluded because tasks were performed in an incorrect order. Data from the remaining 114 participants (105 females, Mean age = 19.45, Age range = 18–28) were analyzed. Participants received a financial compensation or course credits for their participation. Procedures were in accordance with the latest version of the declaration of Helsinki and approved by the local ethics committee (Institute of Psychology, Leiden University).

### Tetris Manipulation

Tetris is a computer game originally designed by Alexey Pajitnov (1984), and exists in many versions. The Tetris game used in the present experiment (Tetris Classic, A. Pajitnov, 1992) can be played individually or together with another person, either in a cooperative or competitive mode. The game consists of a grid in which pieces of a different configuration of four adjacent squares fall down at a constant speed. Falling pieces can be moved left or right with the arrow keys and rotated in intervals of 90°. Pressing the down arrow key increases falling speed. The aim of the game is to fill rows at the bottom of the grid with squares. Each row completely filled with squares will disappear and results in a certain amount of points. The game ends when the stack of squares reaches the top of the grid. The speed by which pieces fall down increases for each level, and a new level starts after 10 rows have been completed. In both the cooperative and competitive condition participants played the game on the same computer within the same grid, with pieces falling down independently from two locations (left and right) at the top of the grid. The participant sitting on the left side of the keyboard controlled the pieces falling on the left side, while the other participant controlled the pieces falling on the right side. Pieces could be moved anywhere within the grid, and both participants could collect points by fitting their own pieces and completing rows at the bottom of the grid. In the cooperative condition participants had to earn as many points as possible together, in this way stimulating cooperative game play. In the competitive condition participants had to earn as many points as possible for themselves, which stimulated competitive game play. In the solo condition participants played Tetris individually sitting next to each other but each on their own computer and independent of the other participant. In the solo condition participants also received points for each row fully filled with squares. Earning points was solely used to stimulate cooperative, competitive, or individual game play, and had no further consequences Importantly, this setup ensured that it was not possible to ignore the actions of the co-actor and thus attention to these was matched during cooperative and competitive game play.

### Task

**FIGURE 1:**
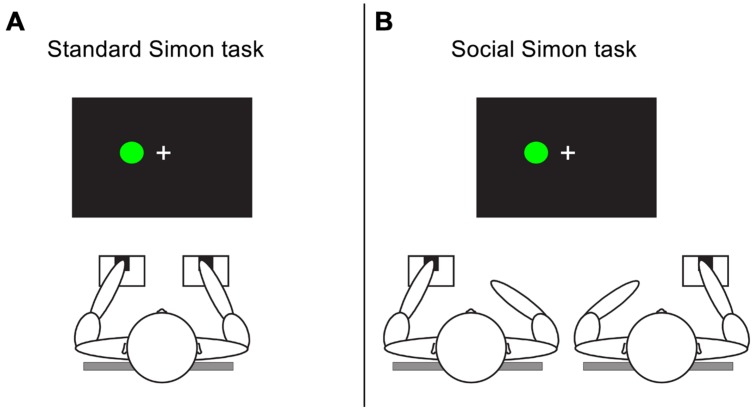
**A schematic representation of the tasks.** In the standard Simon task **(A)** participants perform the task individually and respond to both red and green stimuli using left and right button presses. In the social Simon task **(B)** participants perform the task jointly responding each to one of the two colors with one response button.

A version of the Simon task was used in which red and green circles of 2 cm in diameter were presented 4.5 cm to the left or right of a fixation cross (see, e.g., Colzato et al., 2013; Ruissen and de Bruijn, 2015). Participants responded to the color of the stimulus by pressing the “z”-key on a computer keyboard in response to red stimuli, and the “m”-key in response to green stimuli. Trials consisted of a 500 ms fixation cross followed by the stimulus presented for 1500 ms or until a response had been made. Intervals between subsequent trials were varied randomly between 1000 and 1500 ms in steps of 100 ms. The task consisted of 256 trials (128 compatible and 128 incompatible) divided over four blocks. The color and location of stimuli were random and counterbalanced. In the standard Simon task participants performed the task alone (responding to both types of stimuli). In the social Simon task two participants performed the task together, the left participant responded to red stimuli and the right participant responded to green stimuli (See **Figure 1**).

### Design and Procedure

A counterbalanced mixed between- and within-subject design was used. Participants were assigned to one of the three between-subject conditions (cooperative, competitive, solo). Depending on the assigned condition they first played 8 min of either the cooperative, competitive, or solo version of the Tetris game. This was followed by the standard Simon task or the social Simon task (within subjects). Next, participants played another 8 min of the same Tetris version, followed by the other Simon task. The sequence in which participants performed the standard and social Simon task was counterbalanced across participants.

### Analyses

Reaction times (RTs) were collected for each participant. The first four trials of each block were not included in the analyses. Moreover, trials with erroneous responses and trials with reaction times below 150 ms and above 900 ms were also excluded (0.07%). The reaction-time cut off scores are based on earlier work from our lab where we used the same criteria in similar speeded choice-reaction time paradigms (see, e.g., de Bruijn et al., 2006, 2008, 2012). In line with previous studies (Sebanz et al., 2005; Hommel et al., 2009), reaction times for the standard and social Simon task were analyzed separately using a 2 × 3 repeated measures ANOVA with compatibility (compatible, incompatible) as within-subject factor and Tetris condition (cooperative, competitive, solo) as between-subjects factor. Significant main effects and interactions were further analyzed with one-tailed *t*-tests because of clear directional hypotheses. The significance criterion was set to *p* < 0.05.

## Results

### Standard Simon Task

Analyses of the standard Simon task revealed a main effect of Compatibility [*F*(1,111) = 230.24, *p* < 0.01, $\eta_{p}^{2}$ = 0.67]. Participants responded faster on compatible (*M* = 430 ms) than on incompatible trials (*M* = 458 ms). There was no main effect of Tetris Condition [*F*(2,111) = 0.29, *p* = 0.75, $\eta_{p}^{2}$ = 0.01] nor a significant interaction between the two [*F*(2,112) = 1.76, *p* = 0.18, $\eta_{p}^{2}$ = 0.03]. See **Table 1** for means and standard deviations.

**Table 1:** Mean reaction times (ms) as function of Tetris condition for the standard Simon task.

	Solo (*n* = 38)	Competitive (*n* = 38)	Cooperative (*n* = 38)
RT compatible	425 (54)	435 (51)	429 (44)
RT Incompatible	458 (56)	463 (48)	453 (39)
Standard Simon effect	33 (21)	28 (20)	24 (20)

*Standard deviation in parentheses.*

### Social Simon Task

**FIGURE 2:**
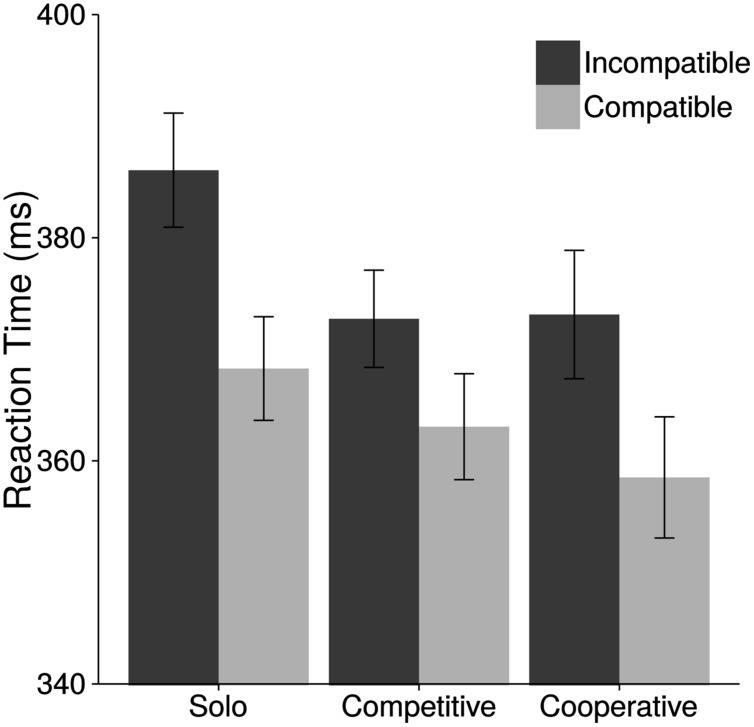
**Mean reaction times for the social Simon task.** Reaction time as function of Tetris condition (solo, cooperative, and competitive) and spatial compatibility. Error bars show standard errors of the mean.

**Figure 2** depicts mean reaction times in the social Simon task as function of Tetris condition and compatibility. Analyses of the social Simon task revealed a significant compatibility effect [*F*(1,111) = 132.63, *p* < 0.01, $\eta_{p}^{2}$ = 0.54] with faster RTs on compatible (363 ms) than on incompatible (377 ms) trials. The main effect of Tetris Condition was not significant [*F*(2,111) = 1.54, *p* = 0.22, $\eta_{p}^{2}$ = 0.03]. However, the interaction between Compatibility and Tetris Condition was significant [*F*(2,111) = 3.81, *p* = 0.03, $\eta_{p}^{2}$ = 0.06]. To further explore this interaction effect, we calculated the SSE by subtracting reaction times on compatible trials from reaction times of incompatible trials. We found significant SSEs in all three conditions [Solo: 18 ms, *t*(37) = 7.66, *p* < 0.001; Competitive: 10 ms, *t*(37) = 5.84, *p* < 0.001; Cooperative: 15 ms, *t*(37) = 6.19, *p* < 0.001]. Importantly, the SSE was smaller following a game of competitive Tetris compared to the cooperative Tetris condition [*t*(74) = 1.74, *p* = 0.04] and compared to the solo Tetris Condition [*t*(74) = –2.92, *p* < 0.01]. There was no difference in the SSE, however, between the cooperative and the solo condition [*t*(74) = –0.98, *p* = 0.17]. See **Table 2** for mean RTs and standard deviations. Please note that we checked for possible task order effects (standard versus social Simon task) with an Order × Condition × Compatibility ANOVA. There was no main effect of Order [*F*(1,108) = 1.20, *p* = 0.28]. Neither were any of the interactions with Order significant [all *F*’s < 1.93, all *p*’s > 0.150].

**Table 2:** Mean reaction times (ms) as function of Tetris condition for the social Simon task.

	Solo (*n* = 38)	Competitive (*n* = 38)	Cooperative (*n* = 38)
RT compatible	368 (29)	363 (29)	358 (34)
RT incompatible	386 (32)	373 (27)	373 (35)
Social Simon effect	18 (14)	10 (10)	15 (15)

*Standard deviation in parentheses.*

### Errors

More errors were made on incompatible trials (Standard Simon task: 6.9%; Social Simon task: 1.4%) than on compatible trials (Standard: 3.5%; Social: 0.5%) in both Simon tasks [Standard: *F*(1,112) = 89.49, *p* < 0.01; Social: *F*(1,112) = 26.90, *p* < 0.01]. Error rates from both tasks did not differ significantly between the three conditions and the interaction with compatibility was not significant [All *F*’s < 1.8; all *p*’s > 0.17].

## Discussion

The present research showed that self-other integration during neutral joint task performance – as reflected in the SSE – depends on the pre-established cooperative or competitive relationship between two individuals. Although the SSE was present in all three contexts, it was significantly reduced following competitive game play. Playing a cooperative, competitive or solo Tetris game did not affect performance on the Standard Simon task. These findings show that people, in general, share each other’s action representations when jointly performing a task. However, the present results demonstrate that when in a previously established and task-unrelated competitive relationship, people do not integrate the task representations of the co-actor to the same degree as compared to a cooperative relationship.

The finding of general shared action representations is in line with Sebanz et al. (2006) who argued that humans – being social in nature – automatically integrate the task of the co-actor into one’s own task during joint action. Interestingly, the present results show that cooperative or competitive game play prior to joint task performance lead to divergent effects on self-other integration. In contrast to previous studies (de Bruijn et al., 2008; Ruys and Aarts, 2010; Iani et al., 2011) we manipulated the relationship prior to a social Simon task, such that no additional cooperative or competitive instructions or changes to the paradigm were needed in the social Simon task. Therefore, the current results can importantly not have been modulated by possible performance differences following from such manipulations. The finding of reduced self-other integration following competitive game play suggests that the amount of self-other integration depends on the pre-established relationship between two individuals, which is in line with suggestions based on fMRI data (Decety et al., 2004) and from social dilemma games (De Cremer and Stouten, 2003).

The SSE emerges when both ones’ own and others’ actions are represented (Sebanz et al., 2003) and overlap exists between the two task representations (Dolk et al., 2014). A smaller SSE when people are in a competitive relationship could then either be explained by less integration of the co-actors action representation or by reduced perceived similarity between ones’ own and the others’ action representations. A reduced SSE in the competitive condition therefore suggests that people in a competitive relationship may not integrate the task of a co-actor to the same degree, or alternatively, may as a consequence of the competitive relationship see themselves and their actions as less similar to the actions of the other person. The latter of the two explanations is in line with studies showing that self-other integration depends on the perceived similarity between actor and co-actor, as people show more task integration when co-acting with a more human like agent (Müller et al., 2011a; Stenzel et al., 2014).

Comparable findings of an attenuated or even absent SSE following a competitive induction were recently published by Iani et al. (2014). In their study, the social Simon task was performed before and after a flanker task in which monetary rewards were used to induce cooperation and competition. They found a regular SSE before the manipulation and following cooperation but the SSE was absent following competition. Alternatively, these results may also be explained by attentional processes induced by the manipulation. When having to compete against another person, people may actually disengage from the co-actor and as a result be more focused on their own task. This may happen for example when people are not motivated to compete or give up during the competition or when it is actually beneficial for their performance not to integrate the task of the co-actor. The latter was shown in a study by de Bruijn et al. (2008) where they found that successful competitors on a competitive task were able to refrain from attending to the other task share. Such differences in attentional processes between the conditions might serve as an alternative explanation for the findings of Iani et al. (2014). In their competitive induction, in which the individual performance in a flanker task is rewarded, a shift toward increased focus on one’s own task and ignoring the task of the co-actor is beneficial for successful performance. Enhanced and sustained focus on one’s own task in the competitive induction task, may thus explain the absence of a SSE on a subsequent social Simon task. Importantly, however, in our Tetris game, attending to the other’s task share and knowing the intentions of the co-actor, is equally important for successful performance in both the cooperative and competitive conditions. Our Tetris manipulation thus induced a cooperative or competitive state without simultaneously and directly influencing attentional processes. It should be noted, however, that even though our manipulation did not affect attention directly, attentional processes may still mediate the relationship between competition and self-other integration as being in a competitive state may generally narrow one’s attention to one’s own task performance.

At a neurochemical level, neuromodulators, including serotonin and oxytocin, plays a pivotal role in social interactions and social relationships (e.g., Lucki, 1998; Bartz and Hollander, 2006). Pharmacological studies have shown that the neurotransmitter serotonin is related to cooperative and competitive behavior. For instance, administration of a selective serotonin reuptake inhibitor (SSRI) facilitates cooperative behavior (Knutson et al., 1998; Tse and Bond, 2002), while depletion of tryptophan, the precursor of serotonin, reduces cooperative behavior (Wood et al., 2006). Similarly, research has shown that administration of the hormone oxytocin enhances cooperative behavior (Declerck et al., 2010) although the effects are context-dependent (Declerck et al., 2014). One explanation for the current findings is that during cooperative game play these neuromodulators are released and may thus have a sustained effect on self-other integration. In support of this, oxytocin-induced enhancement of self-other integration was recently demonstrated in our lab (Ruissen and de Bruijn, 2015). Individual differences in self-other integration, for example as evident in the ability to not integrate (see de Bruijn et al., 2008) may also be related to differences in availability or transportation of these neuromodulators. Therefore, further research is needed aimed at identifying the mechanisms underlying reduced self-other integration following competitive induction as well as elucidating the role of (social) neuromodulators in this process.

Our second hypothesis stated that a cooperative relationship would enhance the SSE in the same way as a competitive relationship attenuates the SSE. However, we did not find a larger SSE following cooperative game play compared to the solo condition, which might be explained by humans’ automatic tendency to cooperate (Bowles and Gintis, 2013). It should be noted that our solo condition did not turn out to be an optimal neutral baseline condition. It was different from the cooperative and competitive conditions in that participants had not yet interacted with each other prior to the social Simon task. Being more or less familiar with the other person may as well affect the degree of self-other integration. One would expect familiarity to enhance self-other integration, for example, through increased perceived similarity. Alternatively, a person with whom you have not interacted before within your personal space may also result in more awareness of the other person. In terms of spatial coding (Dolk et al., 2013) a more salient co-actor can thus enhance the SSE independent of self-other integration. Future studies should aim at disentangling the relative influence of these different aspects further. However, the current study importantly shows that even in situations where attentional processes are matched as best as possible (i.e., cooperative versus competitive induction), self-other integration may be modulated depending on the nature of the previously established relationship.

Finally, the current findings show a sustained effect of competition on the social Simon task. No effect of cooperation or competition was found on the standard Simon task, which was performed in an individual setting. This outcome suggests that the cooperative/competitive manipulation currently used only affects self-other integration and not more general task related attentional processes that are reflected in the standard Simon effect. However, we remain cautious in concluding that the findings are exclusive for the social Simon task, as the two settings cannot be directly compared. The individual Simon paradigm is a two choice reaction-time task and the social Simon paradigm is a (one choice) go/no-go reaction-time task making it difficult to directly compare and interpret the resulting reaction-time patterns. To answer the question whether a competitive relationship only affects self-other integration, a design that better enables direct comparisons of individual and social task performance is needed.

To summarize, the present study showed a sustained effect of competitive game play on the SSE when the social Simon task was performed under neutral conditions. These findings suggest that people in a competitive relationship do not integrate the task representations of the co-actor to the same degree as people in a cooperative or neutral relationship. Importantly, these modulating effects cannot be fully explained by attentional processes, as in our manipulation attending to the other’s task share was equally required in both cooperative and competitive game play. Our findings thus show that the established cooperative or competitive nature of the social relationship between two individuals is sufficient to modulate self-other integration, possibly through changes in perceived similarity.

## Author Contributions

All authors listed, have made substantial, direct and intellectual contribution to the work, and approved it for publication.

## Conflict of Interest Statement

The authors declare that the research was conducted in the absence of any commercial or financial relationships that could be construed as a potential conflict of interest. The handling Editor declared a shared affiliation, though no other collaboration, with the authors and states that the process nevertheless met the standards of a fair and objective review.
